# Novel Simple Conjugation Chemistries for Decoration of GMMA with Heterologous Antigens

**DOI:** 10.3390/ijms221910180

**Published:** 2021-09-22

**Authors:** Roberta Di Benedetto, Renzo Alfini, Martina Carducci, Maria Grazia Aruta, Luisa Lanzilao, Alessandra Acquaviva, Elena Palmieri, Carlo Giannelli, Francesca Necchi, Allan Saul, Francesca Micoli

**Affiliations:** GSK Vaccines Institute for Global Health (GVGH), Via Fiorentina 1, 53100 Siena, Italy; roberta.x.di-benedetto@gsk.com (R.D.B.); renzo.x.alfini@gsk.com (R.A.); martina.x.carducci@gsk.com (M.C.); maria-grazia.x.aruta@gsk.com (M.G.A.); lanzilaol@aou-careggi.toscana.it (L.L.); alessandra.x.acquaviva@gsk.com (A.A.); elena.x.palmieri@gsk.com (E.P.); carlo.x.giannelli@gsk.com (C.G.); francesca.x.necchi@gsk.com (F.N.); allan.saul@honorary.burnet.edu.au (A.S.)

**Keywords:** conjugation chemistry, GMMA, OMV, carrier protein, vaccine, glycoconjugate

## Abstract

Outer Membrane Vesicles (OMV) constitute a promising platform for the development of efficient vaccines. OMV can be decorated with heterologous antigens (proteins or polysaccharides), becoming attractive novel carriers for the development of multicomponent vaccines. Chemical conjugation represents a tool for linking antigens, also from phylogenetically distant pathogens, to OMV. Here we develop two simple and widely applicable conjugation chemistries targeting proteins or lipopolysaccharides on the surface of Generalized Modules for Membrane Antigens (GMMA), OMV spontaneously released from Gram-negative bacteria mutated to increase vesicle yield and reduce potential reactogenicity. A Design of Experiment approach was used to identify optimal conditions for GMMA activation before conjugation, resulting in consistent processes and ensuring conjugation efficiency. Conjugates produced by both chemistries induced strong humoral response against the heterologous antigen and GMMA. Additionally, the use of the two orthogonal chemistries allowed to control the linkage of two different antigens on the same GMMA particle. This work supports the further advancement of this novel platform with great potential for the design of effective vaccines.

## 1. Introduction

The Outer Membrane Vesicle (OMV) platform is receiving great attention for the development of vaccines against bacterial pathogens [1,2]. In this context, OMV have also been proposed as a carrier for protein and polysaccharide antigens from pathogens different from those from which the OMV are derived [3,4]. Kesty and Kuhen were the first to demonstrate the possibility of incorporating heterologous proteins into *E. coli* OMV [5]. Since then, several examples have been reported, showing the ability of OMV constructs to elicit humoral immune responses far more effectively than corresponding recombinant antigenic proteins alone [6,7,8,9,10,11]. Additionally, the feasibility of displaying heterologous glycans on OMV has been demonstrated, resulting in glycoengineered OMV (glyOMV) [4] that combine glycan biosynthesis with OMV from *E. coli* laboratory strains with carbohydrates directly linked to lipid A on OMV surface [12,13,14]. OMV-based vaccines are indeed strongly immunogenic, as they combine a multivalent display of the antigen with nanoparticle size and self-adjuvanting properties [15,16].

Heterologous antigens can also be attached post-OMV production, and different strategies have been proposed for the linkage of proteins or polysaccharides to the OMV surface [17,18,19]. It has been shown that OMV decorated with heterologous polysaccharide or protein antigens through these approaches promote enhanced immunogenicity compared to traditional formulations (e.g., recombinant proteins and glycoconjugate vaccines), without negative impact to the anti-OMV immune response [18,20,21].

Genetic manipulation constitutes the simplest way, from a manufacturing point of view, to achieve the display of antigens in OMV. However, successful strategies need to be identified for each specific antigen and each different OMV-producing bacterium. Additionally, expression of high specific antigen levels on OMV can be difficult [2]. Development of a generic OMV with known expression systems could facilitate expression of heterologous antigen [22]; however, in this way, OMV are not employed to target a specific disease per se but only as carrier for the expressed antigens. On the other hand, chemical conjugation can represent an alternative tool for linking different types of antigens, even derived from phylogenetically distant pathogens, to OMV. Decoration of OMV with antigens (proteins or polysaccharides) from a pathogen different from the one the OMV derive makes them an attractive novel carrier for the development of multicomponent vaccines to target multiple diseases. With the chemical approach, the density and orientation of the antigens can be modulated with relative ease, supporting the design of optimal vaccines [23].

Here we describe the development of two generic, simple and widely applicable conjugation chemistries for efficient linkage of protein antigens to Generalized Modules for Membrane Antigens (GMMA) surface. GMMA are OMV released from Gram-negative bacteria mutated to increase spontaneous blebbing and to reduce their potential reactogenicity, usually by modifying their lipid A structure [24,25,26,27,28].

Our aim was to identify simple processes, reduce conjugation steps, in particular avoiding protein antigen derivatization, obtain good conjugation efficiency and target different components (proteins or lipopolysaccharide (LPS)/lipooligosaccharide (LOS)) on GMMA. A Design of Experiment approach was used to identify optimal conditions for GMMA activation before conjugation, resulting in a consistent conjugation processes, independent of the antigen to be linked. Conjugates produced by both chemistries were tested in mice to verify the ability of the resulting conjugates to induce strong response against the heterologous antigen, but also GMMA per se. Additionally, the use of orthogonal chemistries, targeting different components on GMMA, made it possible to control the linkage of two different antigens on the same GMMA particle, supporting the development of multivalent vaccines, covering multiple diseases at the same time.

## 2. Results

### 2.1. Conjugation Chemistry Targeting LPS/LOS on GMMA

**Figure 1:**
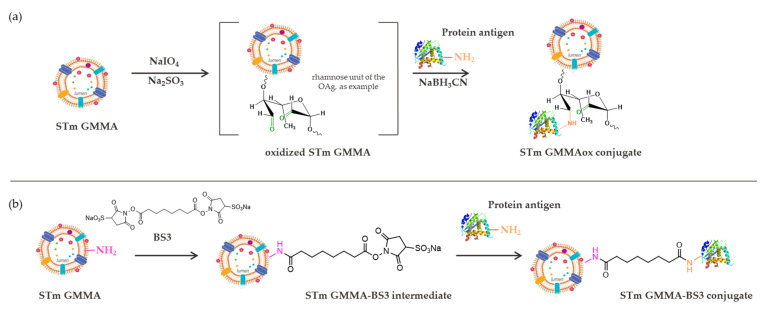
Two novel simple conjugation chemistries developed for decoration of GMMA with heterologous antigens, using STm GMMA as models: (**a**) oxidation of LPS/LOS molecules on GMMA followed by reductive amination with the antigen of interest, (**b**) functionalization of proteins on GMMA with the BS3 linker followed by immediate reaction with the antigen of interest.

The first conjugation strategy we developed involved oxidation of LPS/LOS molecules on GMMA followed by reductive amination with the antigen of interest (Figure 1a). Such procedure can be applied to proteins, through direct linkage of lysine residues. The oxidation step was performed with sodium periodate (NaIO_4_) allowing modification of vicinal diols to generate two aldehyde groups, opening the sugar rings involved [29].

#### 2.1.1. Design of Experiment to Identify the Main Parameters Affecting GMMA Oxidation

To investigate and optimize the GMMA oxidation step, a design of experiments (DoE) approach was used. *S.* Typhimurium (STm) GMMA were selected as model GMMA. The aim was to identify reaction parameters affecting GMMA oxidation and optimal conditions to have good oxidation yield for an efficient subsequent conjugation, preventing major impact on O antigen (OAg) or GMMA structural integrity. Preserving OAg integrity is critical, especially when the OAg is a key target antigen for GMMA immunogenicity, as is the case for STm GMMA [30,31,32]. It is known that OAg oxidation makes the linkages among the sugar units weaker with resulting formation of OAg chains at lower size [33].

A face-centered full factorial response surface design, with one replicate of axial and factorial points and six center point replicates, was used.

Parameters that could affect oxidation step were chosen: GMMA concentration in the range 0.2–4 mg/mL, pH in the range 5–8, and NaIO_4_ concentration in the range 0.5–5 mM. Reaction time and temperature were fixed, respectively, at 30 min and 25 °C, based on preliminary results. Oxidized vesicles were assessed for percentage of GMMA recovery, percentage of GMMA oxidation, GMMA size, and OAg chain length. Conditions used for the oxidation tests are summarized in Table S1.

Similar GMMA recoveries were confirmed for all reaction conditions, as tested by micro bicinchoninic acid protein assay (micro BCA). None of the reaction conditions tested gave rise to GMMA aggregation or degradation, as no changes were observed in terms of particles size distribution by dynamic light scattering (dls).

With a backward elimination process, the non-significant terms (*p*-value > 0.05) were removed and 2 FI models were chosen for either % GMMA oxidation and OAg size (statistical analysis in Figure S1). For % GMMA oxidation, a square root transformation of the response was identified as appropriate by Box-Cox protocol. The residuals, for both models, resulted normally distributed (Anderson-Darling test, *p* = 0.055 for GMMA oxidation and *p* = 0.109 for OAg chain length). For both models, the lack of fit resulted in not being significant (*p* = 0.692 GMMA oxidation and *p* = 0.112 OAg size). The models showed adjusted-R^2^ of 0.99 and 0.94, for GMMA oxidation and OAg size, respectively.

**Figure 2:**
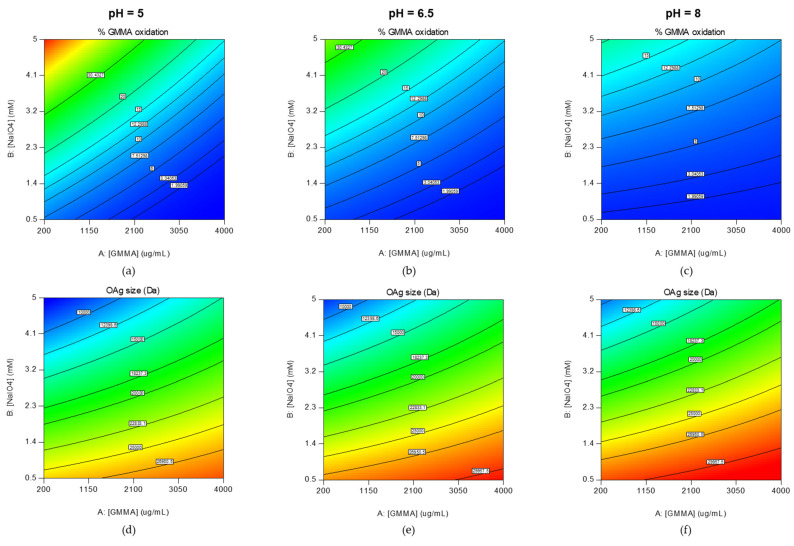
Identification of optimal conditions for GMMA oxidation: 2D surface contour plot for % GMMA oxidation (**a**–**c**) and OAg size (**d**–**f**) responses at pH of 5 (**a**,**d**), 6.5 (**b**,**e**) and 8 (**c**,**f**).

Both % of GMMA oxidation (calculated by High Performance Anion Exchange Chromatography with Pulsed Amperometric Detection; HPAEC-PAD) and OAg apparent molecular weight (estimated by high performance liquid size exclusion chromatography (HPLC-SEC)) were affected by all factors investigated. Lower GMMA and higher NaIO_4_ concentrations resulted in higher oxidation degree (Figure 2). Lower pH increases the oxidation level, mainly at higher NaIO_4_ concentration and with lower GMMA concentration (interaction between factors). As expected, chain length was reduced as % GMMA oxidation progressed.

#### 2.1.2. Impact of Oxidation Degree on GMMA Immunogenicity and Conjugation Efficiency

The reaction conditions of runs 5, 9 and 14 Table S1 were reproduced to verify the impact that different GMMA oxidation degree can have on the immunogenicity elicited by GMMA per se and on conjugation efficiency. For each condition selected, the results were in agreement with those expected according to the models found (Table 1), confirming consistency of the process, as all the responses obtained were within the 95% of tolerance interval (TI) for 99% of population.

Oxidized STm GMMA were tested in mice compared to starting STm GMMA, to investigate the impact of OAg size/oxidation degree on the immune response elicited by GMMA per se.

**Figure 3:**
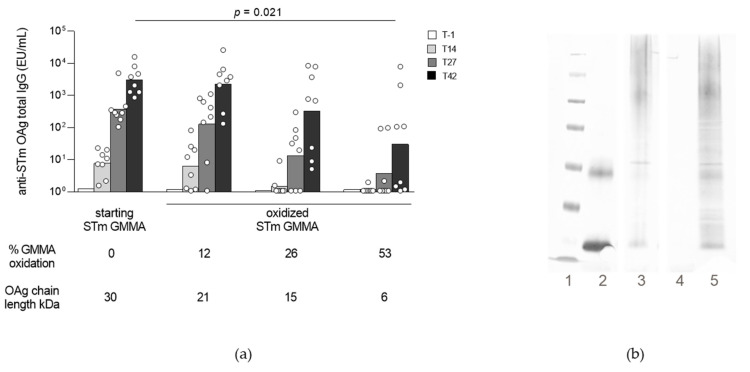
Impact of GMMA oxidation degree on GMMA immunogenicity in mice and conjugation efficiency. (**a**) Immunogenicity of oxidized STm GMMA compared to starting GMMA. CD1 mice were immunized subcutaneously at day 0 and 28 with 0.5 μg mannose dose. All constructs were formulated in saline. Sera were analyzed at days −1, 14, 27 and 42 by enzyme-linked immunosorbent assay (ELISA) using as coating antigen STm OAg. Summary graph of anti-antigen specific IgG geometric mean units (bars) and individual antibody levels (dots) is reported. (**b**) Characterization by WB analysis of the purified conjugates in comparison to corresponding unconjugated Pfs25. Ten µg of conjugates and 2 µg of protein were loaded per well. Lane 1: marker, lane 2: Pfs25, lane 3: conjugate with 26% oxidized GMMA, lane 4: conjugate with 14% oxidized GMMA, lane 5: conjugate with 60% oxidized GMMA.

All GMMA were able to induce specific anti-OAg IgG response, independently from the OAg chain length/oxidation degree. Only GMMA with 6 kDa OAg resulted in induce significantly lower IgG response with respect to starting GMMA (Figure 3a). From this study, we found that an OAg size ≥ 15 kDa with % GMMA oxidation ≤ 26% can be acceptable for preserving GMMA immunogenicity.

In parallel to this study, the same oxidized GMMA (Table 1) were also conjugated to a model protein antigen, in order to assess the impact of GMMA oxidation degree on conjugation efficiency.

Pfs25, a human malarial transmission-blocking protein [34,35], was chosen as antigen to be conjugated to STm GMMA, with the potential to cover both malaria and invasive nontyphoidal salmonellosis, both affecting mainly young children in Africa [36,37,38].

Conjugate formation was verified through sodium dodecyl sulfate-polyacrylamide gel electrophoresis/Western blot (SDS-PAGE/WB) analysis for GMMA oxidized at 26 and 60%, while no conjugate was obtained with 14% oxidized GMMA (Figure 3b). Fourteen percent of GMMA oxidation corresponds to around six oxidized rhamnose (Rha) units per OAg chain, very likely with a corresponding concentration of reactive groups not sufficient for conjugation of Pfs25. Treating Pfs25 with NaBH_3_CN only in order to mimic conjugation conditions in the absence of GMMA, no antigen aggregation and no disulfide bonds reduction were verified, confirming that protein conformation was retained (data not shown).

Considering results obtained from the immunogenicity study in mice and in terms of conjugation efficiency, oxidation conditions of run 9 were chosen as optimal ones for STm GMMA oxidation. Amount of Pfs25 linked in the corresponding conjugate was quantified by competitive enzyme-linked immunosorbent assay (competitive ELISA, cELISA) and resulted in be 27.3% Pfs25/total protein *w*/*w*.

### 2.2. Conjugation Chemistry by Targeting Proteins on GMMA

As an alternative to reductive amination, we developed a second conjugation approach, targeting proteins on GMMA. The conjugation process comprises, first, the functionalization of the proteins on GMMA with the divalent homobifunctional linker Bis(sulfosuccinimidyl) suberate (BS3) and, second, the immediate reaction of the GMMA-linker intermediate with the foreign protein antigen (Figure 1b).

#### 2.2.1. DoE to Identify Optimal Conditions for GMMA Functionalization with BS3

A DoE approach was applied to find the optimal combination of key parameters to increase GMMA activation with BS3, to guarantee efficiency of conjugation, ensuring conjugate formation independently from the antigen to be linked to GMMA. In particular, we aimed to minimize sulfo-NHS ester hydrolysis side reaction, which is critical as it competes with the primary amine reaction contributing to less-efficient conjugation.

A face-centered full factorial response surface design was used. Runs were divided into two blocks, one for axial points and the other for factorial points. Two center point replicates were performed in the axial block and four center point replicates were performed in the factorial block.

STm GMMA were used as model GMMA and parameters that could affect GMMA activation with BS3 were identified: GMMA concentration in the range 0.2–4 mg/mL, pH in the range 6–9 and BS3 equivalents with respect to NH_2_ groups on GMMA in the range 5–15, corresponding to BS3 concentration in the range 1–72 mg/mL. Reaction time and temperature were fixed, respectively, at 30 min and 25 °C, based on preliminary data. Activated GMMA were assessed for GMMA recovery percentage, percentage of activated NH_2_ and percentage of active ester groups introduced (active esters at the terminal end of the linker added could be subjected to the side reaction of hydrolysis). GMMA size was also checked to verify that the use of the homobifunctional linker BS3 does not provide substantial crosslinking of GMMA surface proteins. Conditions used for the activation tests are summarized in Table S2.

Similar GMMA recoveries were obtained for all reaction conditions, as tested by micro BCA, and absence of any crosslinking/aggregation was verified by dls.

With a backward elimination process, the non-significant terms (*p*-value > 0.05) were removed from the models, statistical analysis for % of NH_2_ activation and % of active ester groups are reported in Supplementary Figure S2. The residuals, for both models, were normally distributed (Anderson-Darling test *p* = 0.425 for % of NH_2_ activation and *p* = 0.224 for % of active ester groups introduced). Furthermore, the models resulted with lack of fit of *p* = 0.363 and *p* = 0.356 and with adjusted-R^2^ of 0.97 and 0.72, for % of NH_2_ activation and % of active ester groups respectively.

**Figure 4:**
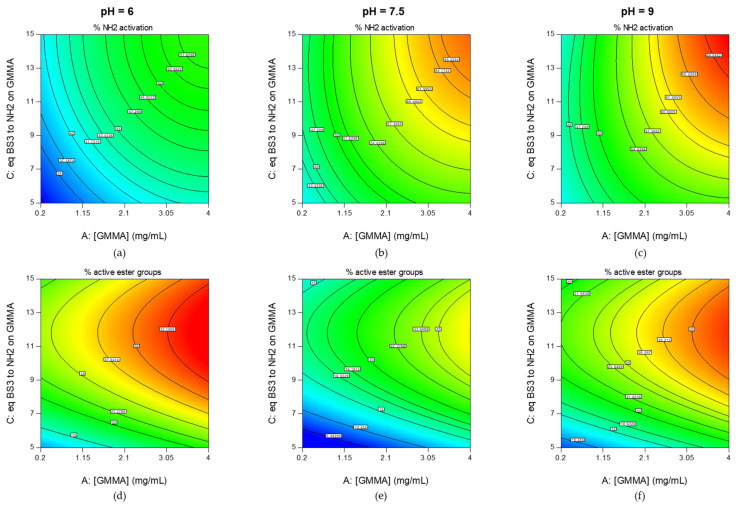
Identification of optimal conditions for GMMA derivatization with BS3: 2D surface contour plot for % NH_2_ activation (**a**–**c**) and % active ester groups (**d**–**f**) responses at pH 6 (**a**,**d**), 7.5 (**b**,**e**) and 9 (**c**,**f**).

For both the responses evaluated, all factors investigated affected the responses (Figure 4). In particular, the % of NH_2_ activated was enhanced at increased GMMA concentrations and at higher BS3 equivalents. This behavior was amplified at high pH. The % of active ester groups introduced was higher at increased GMMA concentrations and BS3 equivalents. The latter was the parameter with the most prominent effect.

Based on the results, optimization was done, with all factors in range, maximizing the % of reactive functional groups introduced with higher importance than for % NH_2_ derivatization maximization.

Optimal conditions identified are reported in Table 2, along with predicted responses and with the actual results obtained by performing the activation in the selected reaction conditions. Results obtained were in agreement with those expected, confirming consistency of the process, as all responses obtained were within the 95% of TI relative to 99% of population.

#### 2.2.2. Impact of % Active Ester Groups on Conjugation Efficiency

Conjugation efficiency was then verified by applying the optimized derivatization conditions with BS3 and using Pfs25 as model antigen to be conjugated to STm GMMA.

**Figure 5:**
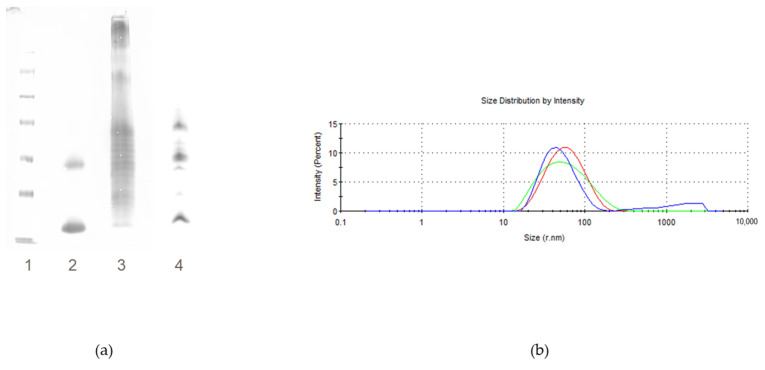
Impact of GMMA-BS3 activation degree on conjugation efficiency. Conjugate characterization. (**a**) WB analysis of the purified conjugates in comparison to corresponding unconjugated protein. Ten µg of conjugates and 2 µg of protein were loaded per well. Lane 1: marker, lane 2: Pfs25, lane 3: conjugate with 32% activated GMMA-BS3, lane 4: conjugate with 11% activated GMMA-BS3. (**b**) dls analysis of GMMA (red line, Z average r of 52.0 nm, PdI of 0.209); GMMA-BS3 (32% activation) (green line, Z average r of 47.1 nm, PdI of 0.245) and GMMA-BS3-Pfs25 conjugate (blue line, Z average r of 49.8 nm, PdI of 0.329 nm), confirming no crosslinking after GMMA derivatization with BS3 linker and conjugation to Pfs25.

Conjugate formation was confirmed by WB analysis (Figure 5a) and analysis by cELISA revealed 19.2% Pfs25 to total protein *w/w* ratio in the purified conjugate. No crosslinking was verified in both the steps of GMMA activation with BS3 and following conjugation, as verified by dls (Figure 5b).

Furthermore, an additional reaction condition was selected and reproduced, resulting according to the model in lower active ester groups introduced (Table 2), to confirm the impact of GMMA activation on conjugation efficiency.

Activated GMMA were conjugated to Pfs25 and WB analysis confirmed the formation of the conjugates (Figure 5a), but with very low smear, as confirmed by cELISA revealing only 3% Pfs25 to total protein *w/w* ratio in the final conjugate. From these results GMMA activation of 30–35% was selected to have efficient conjugation.

### 2.3. Comparison of the Two Conjugation Approaches in Mice

**Figure 6:**
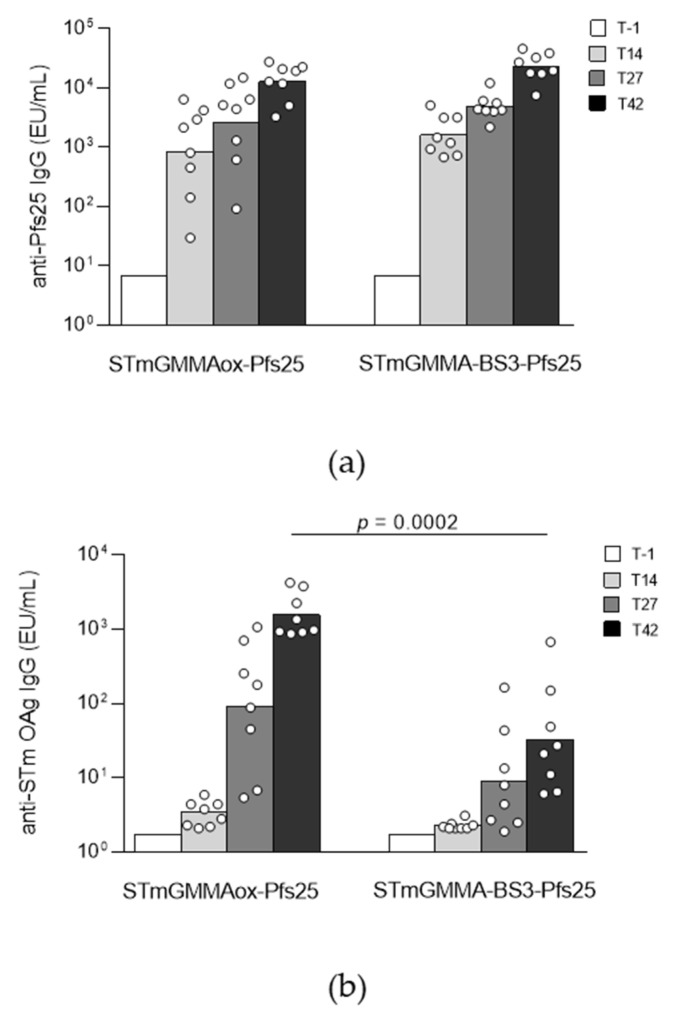
Immunogenicity of Pfs25 GMMA conjugates produced by reductive amination or BS3 chemistry in mice. CD1 mice were immunized subcutaneously at day 0 and 28 with 2.5 μg total protein dose. All constructs were formulated with 0.7 mg/mL Alhydrogel. Sera were analyzed at days −1, 14, 27 and 42 by ELISA using as coating antigens Pfs25 (**a**) or STm OAg (**b**). Summary graphs of anti-antigen specific IgG geometric mean units (bars) and individual antibody levels (dots) are reported.

The two Pfs25-GMMA conjugates produced via reductive amination and BS3 chemistries were compared in mice to check if linkage of Pfs25 to LPS or proteins on STm GMMA could differently impact on the induced humoral immune response. The conjugates were tested at 2.5 μg total protein dose, as both resulted in ~20% linked Pfs25 by cELISA. At all time points tested, the two conjugates induced no significantly different anti-Pfs25 total IgG response (Figure 6a), demonstrating no impact of the conjugation chemistry used. The GMMAox conjugate confirmed the ability to induce a strong anti-OAg IgG response, while the conjugate produced via BS3 chemistry induced reduced anti-OAg IgG response (Figure 6b). Activation with BS3 was performed at pH 9, and we verified a reduction of OAg O-acetylation from 73.4% to 40%. Indeed, O-acetylation level is another parameter, along with OAg chain length, that affects the immunogenicity of STm OAg [39].

### 2.4. Further Improvements of the Conjugation Processes

#### 2.4.1. Conjugation by Reductive Amination: Quenching the Excess of Periodate with Sodium Sulfite

**Scheme 1:**
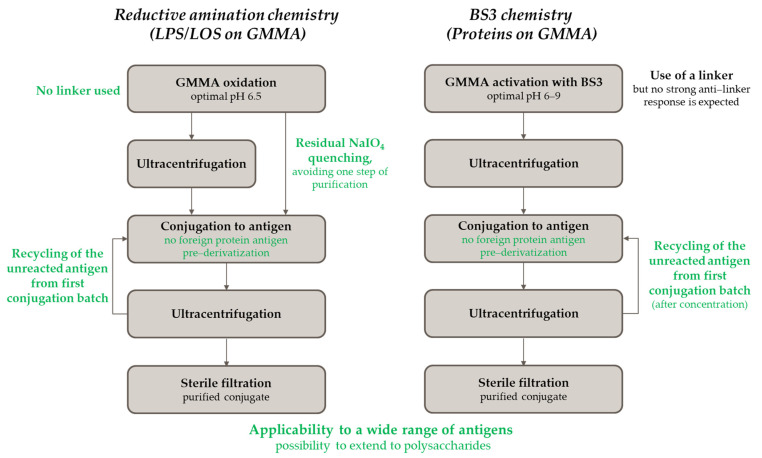
Comparison of the two conjugation approaches developed for linkage of heterologous protein antigens to GMMA surface.

To assess whether the oxidation process could be further simplified (Scheme 1), excess of the oxidizing agent was neutralized with sodium sulfite (Na_2_SO_3_), to perform the process in one step, allowing direct addition of the foreign antigen for conjugation avoiding the isolation of the oxidized GMMA intermediate.

**Figure 7:**
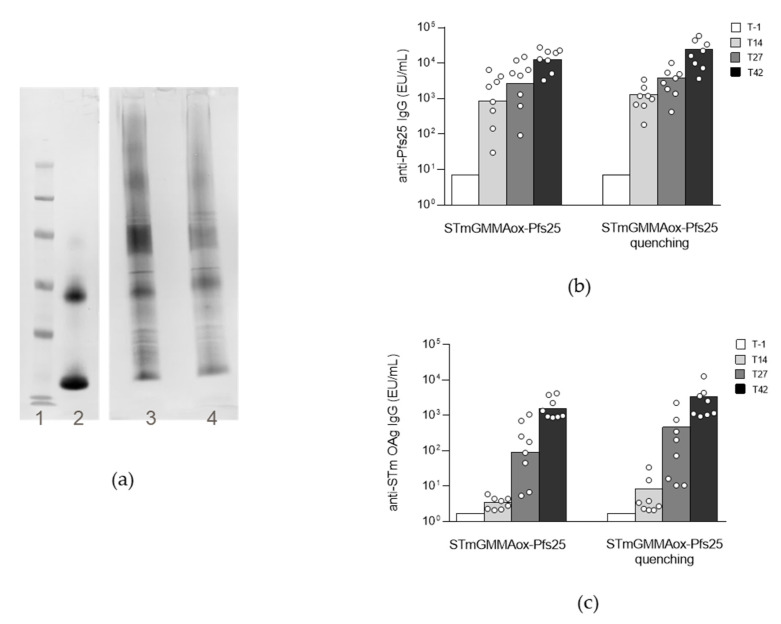
Further simplification of the reductive amination chemistry by introducing a NaIO_4_ quenching step. (**a**) Characterization by WB analysis of the conjugates in comparison to corresponding unconjugated Pfs25. Ten µg of conjugates and 2 µg of protein were loaded per well. Lane 1: marker, lane 2: Pfs25, lane 3: STm GMMAox-Pfs25, lane 4: STm GMMAox-Pfs25 quenched. (**b**,**c**) Comparison in mice of GMMAox-Pfs25 conjugates prepared with or without the quenching step. CD1 female mice were immunized subcutaneously at days 0 and 28 with 2.5 μg total protein dose. Both conjugates were formulated with 0.7 mg/mL Alhydrogel. Sera were analyzed at days −1, 14, 27 and 42 by ELISA using as coating antigens Pfs25 (**b**) or STm OAg (**c**). Summary graphs of anti-antigen specific IgG geometric mean units (bars) and individual antibody levels (dots) are reported.

An STm GMMAox-Pfs25 conjugate was prepared adding the quenching step. WB analysis showed conjugate formation (Figure 7a), with the quenched conjugate having a comparable smear to the conjugate prepared by classical approach. Indeed, the amount of linked Pfs25 to total protein *w/w* ratio was confirmed to be of 20.3% by cELISA, similar to the 27.3% already verified for the classical conjugate.

When tested in mice, at all time points, the two conjugates induced similar anti-Pfs25 IgG response (Figure 7b). Furthermore, the conjugates were able to induce similar anti-OAg IgG response, as indicated in Figure 7c.

Therefore, the quenching step had no impact on the immune response elicited in mice, avoided GMMA oxidation intermediate purification, and allowed further simplification of the overall conjugation process.

#### 2.4.2. Application of Both Chemistries to Additional Antigens and Introduction of a Recycling Step

Both chemistries were applied to additional antigens, to verify their broad applicability. In particular, oxidized STm GMMA were conjugated to Plasmodium R06C, malaria antigen able to elicit high level of transmission blocking antibodies [40,41], and STm GMMA-BS3 to *Neisseria meningitidis* antigen fHbp v2 [42]. Both nontyphoidal *Salmonella* and *Neisseria meningitidis* are prevalent in Sub-Saharan Africa; thus, there is a clinical rationale in developing a vaccine targeting both diseases [10,43,44].

Furthermore, recycling of the unconjugated antigens from a first conjugation batch was tested for both approaches. For the reductive amination chemistry, the recycled protein was added to a new batch of activated GMMA at the concentration it was after recovery, resulting in conjugate formation despite the lower protein concentration (Figure S3). The ratio of R06C to total protein was quantified by cELISA and resulted in be similar in the two conjugates: 7.2% for the first conjugate and 11.1% for the conjugate made with recycled protein. For BS3 chemistry it was instead needed to concentrate the recycled protein to result in conjugate formation. Still a lower smear was obtained for the recycled BS3 conjugate with respect to the first batch (Figure S3).

**Figure 8:**
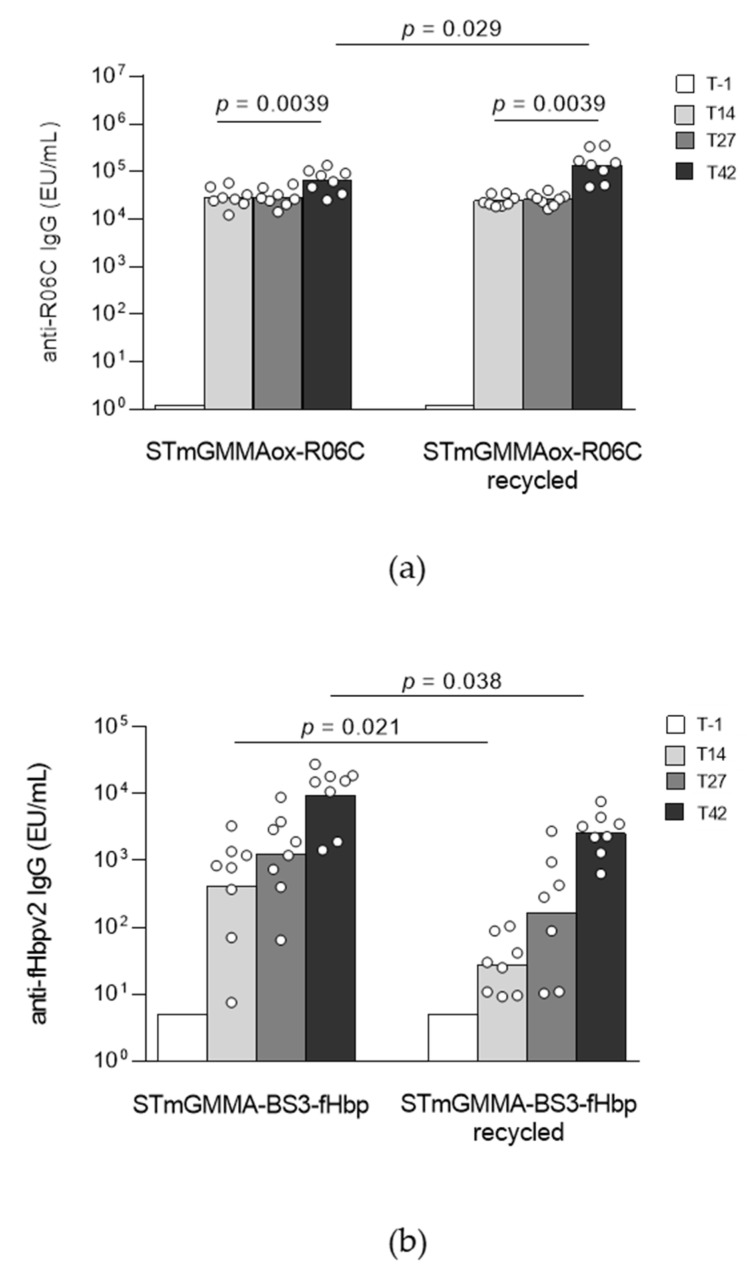
GMMA conjugated to additional heterologous antigens (R06C and fHbp v2) and possibility of recycling the unconjugated protein verified: immunogenicity of R06C conjugates (reductive amination) (**a**) and fHbp v2 conjugates (BS3) (**b**) in mice. CD1 mice were immunized subcutaneously at day 0 and 28 with 4 μg R06C/dose (**a**), or 2.5 μg total protein/dose (**b**). All constructs were formulated with 0.7 mg/mL Alhydrogel. Sera were analyzed at days −1, 14, 27 and 42 by ELISA using as coating antigens R06C (**a**) or fHbp v2 (**b**). Summary graphs of anti-antigen specific IgG geometric mean units (bars) and individual antibody levels (dots) are reported.

When tested in mice, in the case of R06C, both first conjugate batch and recycled conjugate induced high and comparable anti-R06C IgG titers at the different time points tested, with increased response 2 weeks after re-injection (Figure 8a). Similar anti-OAg IgG titers were elicited by both conjugates (Figure S4). For fHbp v2 conjugates, slightly lower anti-fHbp IgG response was induced by BS3 recycled conjugate compared to the non-recycled one, very likely due to the lower amount of linked antigen on GMMA, as in this case the conjugates were compared at the same total protein dose (Figure 8b).

### 2.5. Reductive Amination and BS3 Chemistries for the Introduction of Two Different Proteins on the Same GMMA

The two conjugation approaches developed were used to assess the feasibility of introduction of more than one foreign antigen on the same GMMA, thus resulting in a potential multivalent vaccine [18].

For this purpose, two different *E. coli* antigens, SslE (175 kDa) [45,46] and FdeC (41.7 kDa) [47], were conjugated on the same *S. sonnei* GMMA particle [26], with the potential to protect against two major diarrheagenic pathogens [48].

**Figure 9:**
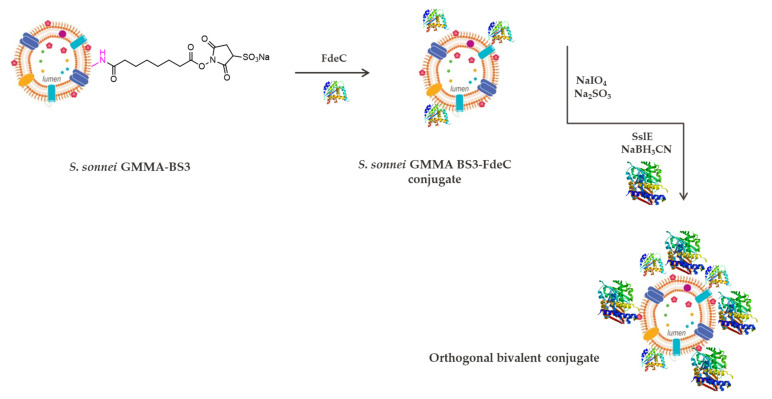
Conjugation strategy used for generation of a bivalent conjugate: conjugation of FdeC on *S. sonnei* GMMA through BS3 chemistry, followed by oxidation and linkage to SslE by reductive amination.

FdeC was conjugated to *S. sonnei* GMMA by BS3 chemistry, followed by GMMA-FdeC oxidation and linkage to SsIE by reductive amination. This orthogonal strategy was selected in order to have the smaller FdeC linked to proteins on GMMA limiting the steric hindrance for the subsequent linkage of SslE to oxidized LPS on GMMA (Figure 9). BS3 chemistry was performed as the first step to ensure linkage of the second *E. coli* antigen to GMMA only.

WB analysis confirmed the presence of both FdeC and SslE antigens on GMMA (Figure S5a). The amount of each conjugated protein was calculated by amino acid analysis [18] and resulted in be: 8.2% for FdeC and 38.7% for SslE. For comparison, corresponding monovalent GMMAox-FdeC and GMMA-BS3-SslE conjugates were generated and resulted in similar loading of the protein antigens on GMMA compared to the bivalent conjugate produced by the orthogonal approach (12% for FdeC and 35% for SslE), indicating that the presence of a first antigen on the GMMA particle does not negatively impact the linkage of a second antigen targeting different moieties on GMMA. No GMMA aggregation was verified by dls for all conjugates (Figure S5b).

The orthogonal conjugate was tested in mice, in comparison to the recombinant proteins alone or singularly conjugated to GMMA particles.

**Figure 10:**
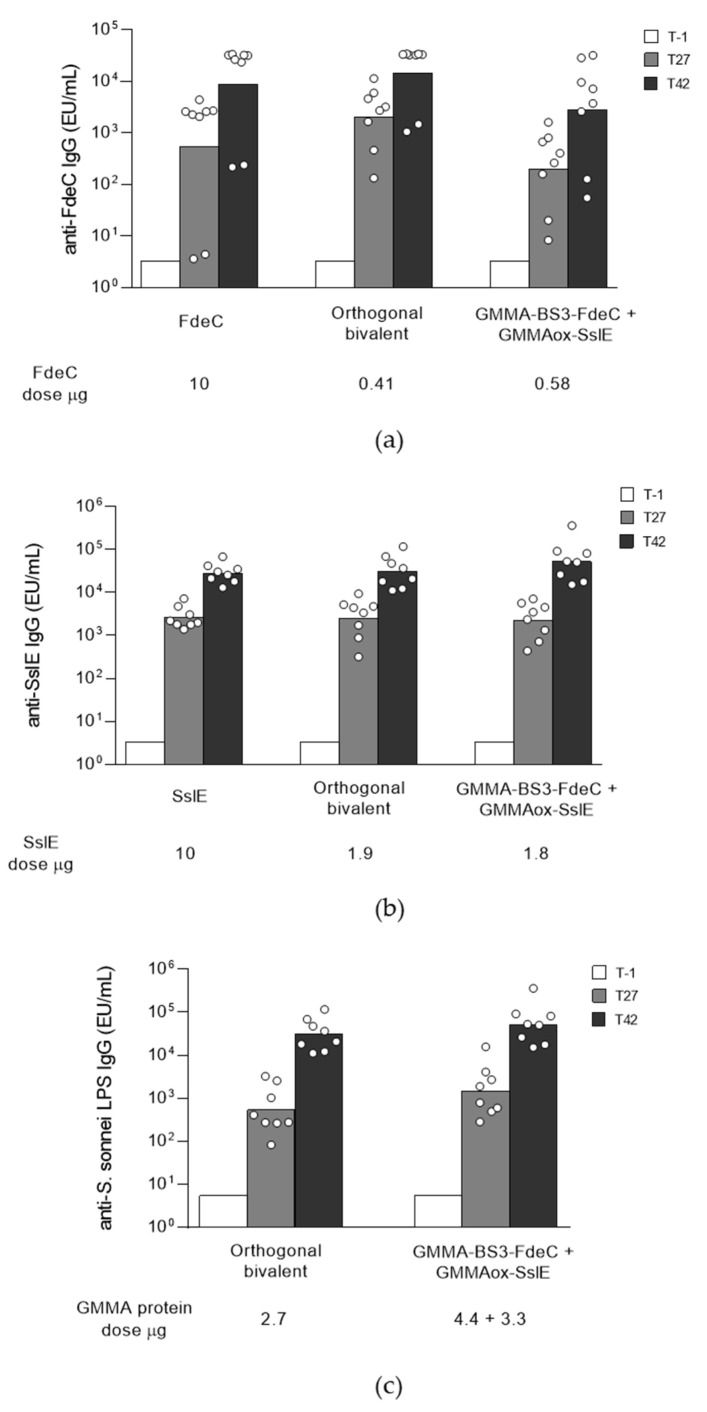
SslE and FdeC presented on the same *S. sonnei* GMMA particle compared to proteins alone and to the corresponding monovalent conjugates mixed together. CD1 mice were immunized intramuscularly at day 0 and 28, with 10 µg *E. coli* protein/dose (group with proteins alone), 5 µg total protein/dose (groups with conjugates). All constructs were formulated with 0.7 mg/mL Alhydrogel. Sera were collected at days −1, 27 and 42 and analyzed by ELISA for anti-*E. coli* antigen-specific (**a**,**b**) and anti-*S. sonnei* LPS (**c**) IgG response. Summary graphs of anti-antigen specific IgG geometric mean units (bars) and individual antibody levels (dots) are reported.

Doses were adjusted to have similar amount of conjugated FdeC and SslE in the two group of conjugates; close to 0.5 μg and 2 μg, respectively. Proteins alone at 10 μg were used as positive control. Interestingly, both monovalent and bivalent conjugates induced anti-*E. coli* antigen specific IgG response comparable to higher amounts (20 times and 5 times for FdeC and SslE, respectively) of the proteins alone. Additionally, the conjugate presenting both *E. coli* antigens on the same particle induced similar anti-*E. coli* proteins IgG response compared to the mixture of the two corresponding monovalent conjugates. Finally, the bivalent conjugate and the mixture of the monovalent ones elicited strong anti-*S. sonnei* LPS IgG titers (Figure 10c).

## 3. Discussion

Decoration of OMV with heterologous antigens can be performed post OMV production through chemical conjugation. Traditional chemistries like carbodiimide adipic acid-mediated [21], click [49,50] and thiol-maleimido chemistries [51] have been proposed.

For example, for conjugation of protein antigens through click chemistry, azido groups are introduced into protein antigens for subsequent coupling to OMV derivatized with alkynes or vice versa. Despite click chemistry having been used for synthesizing many glycoconjugate vaccines [52,53,54,55,56,57], there are some concerns about the stability of azide derivatives, possible immunogenicity of the linker [52,58] and the need for a toxic metal in some applications.

Through thiol-maleimido chemistry, proteins on OMV are derivatized with a linker terminating in a maleimide group that reacts with thiol groups introduced (or natively present) into the antigen, or vice versa, introducing sulfhydryl groups on OMV to react with the antigen activated with maleimide. The sulfhydryl group is a popular target in many modification strategies as, the frequency of sulfhydryl occurrence in proteins or other molecules usually being very low, the use of sulfhydryl-reactive chemistries can restrict modification to only a limited number of sites within a target molecule [59]. However, this chemistry requires immediate reaction with maleimide-modified antigen to avoid loss of reactive thiols due to intramolecular disulfide formation.

All these approaches involve several steps, as they need OMV and heterologous antigen derivatization before conjugation.

Furthermore, the SpyTag-SpyCatcher system was proposed for the display of protein antigens on OMV [17,19]. This approach uses a SpyCatcher domain from a *Streptococcus pyogenes* surface protein, which recognizes a 13-amino-acid peptide (SpyTag) resulting in a covalent isopeptide bond that is formed between the side chains of a lysine in SpyCatcher and an aspartate in SpyTag [60]. Ad hoc modification of OMV and heterologous antigen is needed for the application of this methodology.

Here, we developed novel chemistries for the conjugation of heterologous proteins to OMV. In particular, *S.* Typhimurium GMMA were used as a model. GMMA are produced at high yields through simple and robust detergent-free processes [26,61]. The two chemistries developed, reductive amination and BS3 chemistry, do not require heterologous protein antigen derivatization, as conjugation can go directly through their lysine residues. We have already shown that such chemistries can be applied to diverse protein antigens and also to polysaccharides [18]. In the case of polysaccharides, the introduction of an amino/hydrazide linker is needed if no amino groups are already present in its native/synthetic structure. Additionally, the novel chemistries were selected to target different attachment points on GMMA surface, proteins and LOS/LPS. We showed that, independent of the attachment site on GMMA, the resulting conjugates are strongly immunogenic and able to elicit similar levels of anti-foreign protein specific response. Use of orthogonal chemistries opens up the possibility of better preserving the immune response elicited by GMMA according to their key antigens (proteins or LPS). This is important for using GMMA in the dual role of carrier and antigen [62]. The genetic strategies introduced so far more often make use of *E. coli* OMV as a carrier, while through chemical conjugation, it is becoming easier to use OMV from different pathogens supporting the development of multivalent vaccines, as shown here by combining *E. coli* antigens and *Shigella sonnei* GMMA. In the case of polysaccharides, glycoOMV present the sugar chains attached to lipid A portion. Here, we have the possibility of exploring whether linkage of polysaccharides to proteins on GMMA can result in improved immunogenicity and more easily design optimized conjugates [23].

Additionally, we have already shown the possibility of using these two orthogonal chemistries, targeting different functional groups on GMMA, to introduce two different antigens on the same particles. The use of orthogonal chemistries can allow better control for the introduction of each single antigen, with generation of more consistent products. Importantly, the resulting conjugate induced strong antibody response against the two heterologous antigens, and also against GMMA, supporting the development of multivalent vaccines. Here, two *E. coli* proteins were conjugated to GMMA but, based on our experience, the same approach should work for linkage of antigens from different pathogens.

Reductive amination chemistry was further simplified, avoiding oxidized GMMA intermediate isolation. A comparison of the two chemistries developed here is provided in Scheme 1. Both chemistries also have the advantage of not deactivating functional groups of the antigen to be linked during the conjugation step, thereby allowing the recycling of the unreacted heterologous antigen.

Here, a DoE approach was used to identify optimal conditions for GMMA activation. With respect to the traditional one-factor-at-a-time (OFAT) approach, with minimal experimentation and investment of resources, the DoE methodology makes it possible to identify optimal combination of the critical parameters, also considering their interaction, and to model the process in the design space investigated, predicting impact of changes in the critical parameters on the quality of the final product [63,64]. For example, for *S.* Typhimurium, it is well known that the OAg moiety of LPS is the main target of protective immunity [31,32,65,66]. Thus, for *Salmonella* GMMA, as well as for GMMA from other pathogens for which the OAg moiety of LPS is the key target antigen for an effective response, it is important to prevent a major impact on OAg during conjugation to maintain the immune response against GMMA. We verified that by performing BS3 activation at pH 9, the OAg O-acetylation level was impacted, resulting in lower anti-OAg IgG response [30,31,32,39]. Through DoE and the model obtained, it is possible to immediately identify alternative BS3 activation conditions, fixing the pH at 6 still resulting in 30% GMMA activation.

In cases where GMMA from other pathogens is to be used as carrier, a check would be needed to verify if optimal conditions identified here for *S.* Typhimurium GMMA also apply to them.

Once the GMMA are properly activated, we demonstrated the possibility of efficiently cojugating antigens with different structures using standard conjugation conditions, without the need to identify optimized conditions for each novel antigen to be conjugated.

In conclusion, two novel approaches were identified for the display of heterologous antigens on OMV surface, supporting the further advancement of this novel platform with great potential for the design of effective vaccines.

## 4. Materials and Methods

### 4.1. Materials

*S.* Typhimurium GMMA (obtained from Δ*tolR* Δ*PagP* Δ*msbB* 2192 mutant strain) and *S. sonnei* GMMA (obtained from Δ*tolR* Δ*virG* Δ*htrB* 53G mutant strain) were produced and characterized as previously described [26,67]. *Plasmodium falciparum* Pfs25 recombinant proteins (18 kDa) and *P. falciparum* R06C were kindly provided by the Laboratory of Malaria Immunology and Vaccinology (HHS/NIH/NIAID, Bethesda, MD, USA) and the Department of Clinical Biochemistry, Immunology and Genetics, Statens Serum Institut, Copenhagen, Denmark, respectively. *E. coli* SslE, factor adherence *E. coli* (FdEc), *Neisseria meningitidis* factor H binding protein variant 2 (fHbp v2) recombinant proteins (175, 42 and 27 kDa respectively) were provided by GSK Vaccines, Siena, Italy.

The following chemicals were used in this study: sodium phosphate monobasic (NaH_2_PO_4_), sodium phosphate dibasic (Na_2_HPO_4_), sodium cyanoborohydride (NaBH_3_CN), sodium periodate (NaIO_4_), sodium sulfite (Na_2_SO_3_), sodium borohydride (NaBH_4_), *N*-Hydroxysulfosuccinimide (sulfo-NHS), ammonium hydroxide solution (NH_4_OH) (Merck, Darmstadt, Germany), boric acid solution, phosphate buffered saline tablets (PBS) (Honeywell Fluka, Charlotte, NC, USA).

### 4.2. Conjugation Chemistry Targeting LPS/LOS on GMMA

#### 4.2.1. GMMA Oxidation

After optimization of this step through DoE, STm GMMA and *S. sonnei* GMMA 4 mg/mL were oxidized with 5 mM NaIO_4_ in 100 mM phosphate buffer (NaPi) at pH 6.5. The solution was kept at 25 °C in the dark, for 30 min. After that, the oxidized GMMA were purified via ultracentrifugation and used for conjugation. Ultracentrifuge Thermo Fisher Scientific (Waltham, MA, USA) MX 150+ Micro-Ultracentrifuge equipped with Thermo Fisher Scientific (Waltham, MA, USA) S110-AT rotor (K factor = 15) was used. Centrifugation conditions were 30 min at 4 °C, 110,000 rpm using 4 mL PC Thick Walled Tubes (Thermo Fisher Scientific, Cat No 45239) filled with 2 mL of solution. Alternatively, oxidized GMMA were added of Na_2_SO_3_ at a final concentration of 10 mM, kept for 15 min at room temperature (RT) in slight agitation and then used for conjugation with no intermediate isolation. The STm GMMAox not used for conjugation and tested in mice were treated with NaBH_4_ (NaBH_4_ to total Rha of GMMA OAg 3:1 molar ratio), for 2 h at RT by stirring the mixture, in order to reduce the aldehyde groups generated. The purification was done by ultracentrifugation (110,000 rpm, 30 min, 4 °C).

#### 4.2.2. Conjugation via Reductive Amination

Protein antigens were directly added to ultracentrifuged GMMAox (classical approach) or to quenched GMMAox in a *w*/*w* ratio of GMMA to antigen of 1:1. Protein antigen concentration in each reaction, equal to GMMA concentration, was: 2.6 mg/mL for Pfs25 (classical approach), 1.57 mg/mL for Pfs25 (quenched approach), 2 mg/mL for R06C, 1.23 mg/mL for SslE (quenched approach).

In the recycling experiment, R06C recovered after ultracentrifugation from the first conjugation batch was directly added to STm GMMAox (*w*/*w* ratio of GMMA to R06C 1:1 at a GMMA concentration of ~1 mg/mL).

In all reactions NaBH_3_CN (1:1 *w*/*w* ratio with GMMA) was added immediately after the protein antigen. After gently mixing overnight (ON) at RT, the conjugates were purified by ultracentrifugation (110,000 rpm, 4 °C, 1 h) and resuspended in PBS.

### 4.3. Conjugation Chemistry by Targeting Proteins on GMMA

#### 4.3.1. GMMA Activation with BS3

After optimization of this reaction through DoE, STm GMMA and *S. sonnei* GMMA were activated with BS3 linker at a protein concentration of 4 mg/mL in 100 mM borate buffer, at pH 9. The solution was added to the linker at a final concentration of 50 mg/mL in the reaction mixture. The mixture was kept at 25 °C for 30 min, then activated GMMA were purified by double ultracentrifugation (110,000 rpm, 16 min, 4 °C).

#### 4.3.2. Conjugation via BS3 Chemistry

Protein antigens were immediately directly added to ultracentrifuged GMMA-BS3, always in *w*/*w* ratio of GMMA to antigen of 1:1. Protein antigen concentration in each reaction, equal to GMMA concentration, was: 2.6 mg/mL for Pfs25, 0.92 mg/mL for fHbp v2, 5.75 mg/mL for FdeC.

For the experiment of recycling, fHbp v2 recovered from the first conjugation batch and concentrated via Amicon Ultra (Merck, Darmstadt, Germany) 10 kDa cut-off against PBS (3500 rpm; 4 °C; 4 washes) at ~1 mg/mL was immediately added to ultracentrifuged STm GMMA-BS3 in PBS (*w*/*w* ratio of GMMA to fHbp v2 1:1 at a GMMA concentration of ~1 mg/mL). After gently mixing ON at RT, all conjugates were purified by ultracentrifugation (110,000 rpm, 1 h, 4 °C) and resuspended in PBS.

### 4.4. Synthesis of the Bivalent Conjugate

The orthogonal bivalent conjugate was synthesized as previously described [18]. Briefly, FdeC was first conjugated to *S. sonnei* GMMA-BS3 (*w*/*w* ratio GMMA to FdeC of 1:1, GMMA concentration of 6.45 mg/mL). After purification via double ultracentrifugation (110,000 rpm, 16 min, 4 °C), the FdeC conjugate was oxidized according to the protocol optimized for GMMA and, after quenching with Na_2_SO_3_, SslE (*w*/*w* ratio GMMA to SslE of 1:1, GMMA concentration of 1.18 mg/mL) and NaBH_3_CN were added. After purification via ultracentrifugation (110,000 rpm, 1 h, 4 °C) the final bivalent conjugate was resuspended in PBS.

The non-orthogonal conjugate was prepared by activating *S. sonnei* GMMA with BS3 linker as previously described. After double ultracentrifugation (110,000 rpm, 16 min, 4 °C), both FdeC and SslE were added. Each protein was added at 0.5 to 1 *w*/*w* ratio with respect to GMMA-BS3 (GMMA concentration of 3.0 mg/mL). The reaction mixture was gently stirred ON at RT and after ultracentrifugation (110,000 rpm, 1 h, 4 °C) the conjugate was resuspended in PBS.

### 4.5. Design of Experiment (DoE)

Experimental planning and data elaboration were performed with Design-Expert 10 (Stat-Ease Inc., Minneapolis, MN, USA), Anderson-Darling normality test was performed using Minitab 18, Minitab Inc. (State College, PA, USA).

For GMMA oxidation reaction, conditions detailed in Table S1 were used and purification was done through ultracentrifugation (110,000 rpm, 16 min, 4 °C). After resuspension in PBS all oxidized GMMA were assessed for % GMMA recovery, GMMA size, % GMMA oxidation, OAg chain length.

For GMMA activation with BS3, conditions detailed in Table S2 were used and purification was done through ultracentrifugation (110,000 rpm, 16 min, 4 °C). All GMMA-BS3 were resuspended in PBS and analyzed for % GMMA recovery, GMMA size, % NH_2_ activated and % active ester groups introduced.

For both the DoE tests, the analyses were performed following the same randomization order used to carry out the reactions.

### 4.6. Analytical Methods

Both oxidized GMMA and GMMA-BS3 were characterized by micro BCA (Thermo Fisher Scientific, Waltham, MA, USA) for GMMA recovery and dls for particle size determination [67]. Percentage of GMMA oxidation was verified by HPAEC-PAD after performing acid hydrolysis directly on GMMA for OAg sugar content quantification, as previously described [68], and comparing the OAg Rha to mannose molar ratios before (start) and after (ox) the oxidation step. Indeed, in the case of STm OAg, oxidation can impact Rha and glucose present in the OAg repeat and sugars in the core region of the LPS molecules. Here, for simplicity, and considering that Rha is one of the monomers mainly impacted by the oxidation (diol in cis), oxidation % was calculated only considering the impact on Rha monomers. Mannose instead is not impacted by the reaction as it does not have diols which are target for NaIO_4_ oxidation (Figure S6). The following equation was used, with all concentrations expressed as µmol/mL:

$$\%\;\mathrm{GMMA}\;\mathrm{oxidation}=\left(1-\frac{\left[{\mathrm{Rha}}_{\mathrm{ox}}\right]}{\left(\frac{\left[{\mathrm{Rha}}_{\mathrm{start}}\right]}{\left[{\mathrm{Man}}_{\mathrm{start}}\right]}\right)\times \left[{\mathrm{Man}}_{\mathrm{ox}}\right]}\right)\times 100$$

OAg chain length of GMMAox was determined by HPLC-SEC analysis after OAg extraction through acetic acid hydrolysis, as previously described [24]. GMMA-BS3 were assessed for % NH_2_ activation through 2,4,6-trinitrobenzene 1-sulfonic acid assay (TNBS) [69] and % active ester groups introduced by HPLC-SEC, as described below.

GMMA conjugates were characterized by micro BCA for total protein recovery and SDS-PAGE/WB analysis to confirm conjugate formation, as previously described [18]. Anti-Pfs25 monoclonal antibody 4F7 provided by the Laboratory of Malaria Immunology and Vaccinology (HHS/NIH/NIAID, Bathesda, MD, USA), anti-R06C monoclonal antibody anti pfs45/48 epitope 1 provided by the Department of Clinical Biochemistry, Immunology and Genetics, Statens Serum Institut (Copenhagen, Denmark), polyclonal sera internally generated in mice against fHbp v2, FdeC and SslE were used as primary antibodies.

For Pfs25 and R06C, the amount of linked protein antigen was determined via cELISA [70,71] as detailed below. For FdEc and SslE, quantification through amino acid analysis was performed, as described in [18]. The estimation of the eventual presence of residual unconjugated proteins was done by analyzing the purified conjugates through HPLC-SEC by using TSK gel 4000–6000 PW columns (TOSOH, Tokyo, Japan) in series and eluting with PBS for 60 min at a flow rate of 0.5 mL/min [67].

#### 4.6.1. High-Performance Liquid Chromatography–Size Exclusion Chromatography (HPLC-SEC) to Determine % Active Ester Groups Introduced

Active esters present in ultracentrifuged GMMA-BS3 samples were hydrolyzed with ammonium hydroxide solution (NH_4_OH) at 0.085% final concentration for 1 h stirring at RT, in order to release the *N*-Hydroxysulfosuccinimide (sulfo-NHS) ester groups. Samples were then evaporated to dryness using a centrifugal evaporator like SpeedVac (Thermo Fischer, Waltham, MA, USA). Hydrolyzed samples were then redissolved in water and analyzed in comparison to a sulfo-NHS calibration curve prepared by serial diluting sulfo-NHS reagent in water.

The analysis was performed eluting all samples on a Shodex OHpak SB-802.5 HQ (8.0 × 300 mm) column (particle size 6 µm) (Showa Denko America, Inc., New York, NY, USA). The mobile phase was PBS at the flow rate of 1.0 mL/min (isocratic method for 25 min). Sample volume of injection was 80 µL. Sulfo-NHS ester groups were detected at 260 nm.

Percentage of active ester groups introduced was calculated using the following equation, with sulfo-NHS and total NH_2_ concentrations expressed as µmol/mL and GMMA concentrations expressed as µg/mL:

$$\%\;\mathrm{active}\;\mathrm{ester}\;\mathrm{groups}=\left(\frac{\frac{\left[\mathrm{sulfo}-\mathrm{NHS}\right]}{\left[\mathrm{GMMA}-\mathrm{BS}3\right]}}{\frac{\left[{\mathrm{GMMA}}_{\mathrm{start}}{\;\mathrm{total}\;\mathrm{NH}}_{2}\right]}{\left[{\mathrm{GMMA}}_{\mathrm{start}}\right]}}\right)\times 100$$

#### 4.6.2. Competitive ELISA (cELISA)

cELISA assay was performed as previously described [70,71]. A standard curve was prepared by 10 sequential 2-fold or 1.4-fold dilution steps of protein antigens for Pfs25 or R06C respectively, starting from a known concentration (1.2 µg/mL for R06C and 120 µg/mL for Pfs25). STm GMMA conjugates were tested as undiluted (starting concentration in total protein in a range of 25–50 µg/mL) and then in 5 or 7 sequential 2-fold dilutions. Standard curve and test samples were diluted in ELISA Sample Dilution Buffer (PBS with 0.1% BSA and 0.5% Tween-20). Anti-protein monoclonal antibodies (mAbs) were diluted in PBS with 1% BSA and 0.5% Tween-20 and then added to the diluted samples in a ratio 1:1 (Pfs25 and R06C mAbs final dilution 1:10,000 and 1:1000 respectively). All mixtures were pre-incubated overnight (ON) at 4 °C shaking. Each assay was run in duplicate on the same plates (Nunc round bottom Maxisorp ELISA plates, Thermo Fisher Scientific, Waltham, MA, USA). Plates were coated ON at 4 °C with protein at 1 µg/mL in carbonate buffer pH 9.6. The coating solution was removed, and plates blocked with 5% fat-free milk dissolved in PBS buffer. After 1 h incubation at 25 °C, plates were washed three times with washing buffer (PBS with 0.05% Tween-20). The pre-incubated mixtures of samples/standard curve with mAb were added to each ELISA plate, and the plates were incubated for 2 h at RT (about 21 °C) in a plate shaker (MixMate from Eppendorf AG, Hamburg, Germany) set at 600 rpm. Plates washing step was then repeated and a secondary antibody anti-mouse IgG conjugated to alkaline phosphatase (A3438, Merck, Darmstadt, Germany) was added to the plates and incubated for 1 h at 25 °C. After three more washes in a washing buffer, 100 µL of p-nitrophenyl phosphate substrate solution (N2770, Merck, Darmstadt, Germany) were added and plates were incubated for 1 h at 25 °C. Absorbance was read at 405 nm and 490 nm and the difference between them (OD405nm-OD409nm) was measured. The standard curve was fitted with a four-parameter logistic curve model using GraphPad Prism 6 software (GraphPad Software, La Jolla, CA, USA), and the amount of protein antigen in the test samples was calculated by nonlinear regression analysis.

This assay was optimized for Pfs25 and R06C, while no consistent results were obtained for fHbp conjugates. The method does not provide an absolute quantification of the protein antigen linked as the behavior of the free protein with respect to the conjugated protein in the recognition of the mAb could be different, but allowed us to compare relative amount of conjugated proteins in the conjugates.

### 4.7. Immunogenicity Studies in Mice

Immunization experiments were performed at the GSK Animal Facility in Siena or at Toscana Life Sciences Animal Facility (Siena, Italy), in compliance with the relevant guidelines (Italian D. Lgs. n. 26/14 and European directive 2010/63/UE) and the institutional policies of GSK. The animal protocols were approved by the Animal Welfare Body of GSK Vaccines, Siena, Italy, the Animal Welfare Body of Toscana Life Sciences, Siena, Italy and the Italian Ministry of Health (Approval numbers 804/2015-PR, 479/2017-PR).

Female, 5-week-old CD1 mice (8 per group) were vaccinated subcutaneously (s.c.) or intramuscularly (i.m.) at days 0 and 28. Approximately 100 µL bleeds (50 µL serum) were collected at day −1 (pooled sera), 14 and 27 (individual sera) with final bleed at day 42. Conjugates were formulated with 0.7 mg/mL Alhydrogel (Al^3+^). By SDS-PAGE silver staining analysis it was verified, by comparing lanes loaded with Alhydrogel supernatant and the conjugate at 10% of total dose, that >90% of the conjugates was adsorbed on Alhydrogel.

Anti-antigen-specific IgG levels were measured at days −1 (pooled sera), 14, 27 and 42 by ELISA [30]. Purified Pfs25, R06C, fHbp v2, SslE and FdeC proteins were used for ELISA plate-coating at 1 µg/mL in carbonate buffer or PBS buffer (the latter for SslE and FdeC). Purified OAg from *S*. Typhimurium was used for ELISA plate-coating at 5 µg/mL in carbonate buffer, purified *Shigella sonnei* LPS was used at 0.5 µg/mL in PBS. ELISA units were expressed relative to a mouse antigen-specific antibody standard serum curve, with the best five-parameter fit determined by a modified Hill plot. One ELISA unit is defined as the reciprocal of the dilution of the standard serum that gives an absorbance value equal to 1 in this assay. Each mouse serum was run in triplicate.

### 4.8. Statistics

Mann–Whitney two-tailed test was used to compare the immune response elicited by two different antigens at same time point, while Wilcoxon test matched-pairs signed rank one-tailed test was performed to compare the response induced by the same antigen at day 14 vs. day 42.

## Figures and Tables

**Table 1:** Three different conditions selected for producing GMMA characterized by different GMMA ox % and OAg chain length, with expected results confirmed by experimental ones.

Run	Conditions for Oxidation			Expected GMMA ox % (95% TI)	Expected OAg Size Da (95% TI)	I Set Experiments (Used for Study in Mice)		II Set Experiments (Used for Conjugation Tests)	
	[GMMA] mg/mL	[NaIO_4_] mM	pH			GMMA ox %	OAg Size Da	GMMA ox %	OAg Size Da
5	0.2	5	5	57.1 (42.2–74.1)	6200 (2211–14,610)	53	6039	60	7038
9	2.1	5	6.5	23 (15.1–32.2)	12,266 (4794–19,738)	26	15,327	26 ^1^	15,327 ^1^
14	2.1	2.75	6.5	9.1 (4.7–14.8)	20,729 (13,733–27,725)	12	21,163	14	23,464

^1^ For run 9 the oxidation was performed once, and the same lot was used either for study in mice and for conjugation tests.

**Table 2:** Optimized conditions for GMMA activation with BS3 with expected responses from the model, confirmed by performing an additional test, and activation conditions selected for assessing impact of % of active ester groups on conjugation efficiency.

Run	Conditions for GMMA-BS3 Activation			% Active Ester Groups Introduced	
	[GMMA] mg/mL	[BS3] mg/mL (eq Respect to NH_2_ on GMMA)	pH	Expected (95% TI)	Obtained
Optimized condition	4	50 (10.4)	9	31.3 (11.3–51.3)	32
1	0.2	0.2 (5)	6	10.6 (0–30.2)	11

## Data Availability

Data is contained within the article or Supplementary Materials.

## Supplementary Materials

The following are available online at https://www.mdpi.com/article/10.3390/ijms221910180/s1.
