# Supplementary material for: Novel Simple Conjugation Chemistries for Decoration of GMMA with Heterologous Antigens
Source: Int J Mol Sci. 2021 Sep 22;22(19):10180. doi: 10.3390/ijms221910180 (PMC8508390; doi:10.3390/ijms221910180)
Supplement: Supplementary file 1 [file ijms-22-10180-s001.zip › ijms-1382583-supplementary.pdf]

Supplementary Materials

Novel simple conjugation chemistries for decoration of GMMA with heterologous antigens

Roberta Di Benedetto [1], Renzo Alfini [1], Martina Carducci [1], Maria Grazia Aruta [1], Luisa Lanzilao [1#], Alessandra Acquaviva [1], Elena Palmieri [1], Carlo Giannelli [1], Francesca Necchi [1], Allan Saul [1†] and Francesca Micoli [1,*]

1 GSK Vaccines Institute for Global Health (GVGH), Via Fiorentina 1, 53100 Siena, Italy; roberta.x.di-benedetto@gsk.com (R.D.B.); renzo.x.alfini@gsk.com (R.A.); martina.x.carducci@gsk.com (M.C.); maria-grazia.x.aruta@gsk.com (M.G.A.); lanzilaol@aou-careggi.toscana.it (L.L.); alessandra.x.acquaviva@gsk.com (A.A.); elena.x.palmieri@gsk.com (E.P.); carlo.x.giannelli@gsk.com (C.G.), francesca.x.necchi@gsk.com (F.N.), allan.saul@honorary.burnet.edu.au (A.S.)

Was GSK employee at the time of the study was performed, currently General Laboratory, AOU-Careggi, 50139 Firenze, Italy

† Was GSK employee at the time of the study was performed, currently Burnet Institute, Melbourne, Australia

* Correspondence: francesca.x.micoli@gsk.com (F.M.); Tel.: +39 346 2517893

Table S1. DoE approach applied to GMMA oxidation: summary of conditions tested.

Std	Run	Factor 1 A:[GMMA] mg/mL	Factor 2 B:[$NaIO_4$] mM	Factor 3 C:pH
19	1	2100	2.75	6.5
14	2	2100	2.75	8
2	3	4000	0.5	5
13	4	2100	2.75	5
3	5	200	5	5
18	6	2100	2.75	6.5
9	7[1]	200	2.75	6.5
10	8	4000	2.75	6.5
12	9	2100	5	6.5
16	10	2100	2.75	6.5
11	11	2100	0.5	6.5
1	12	200	0.5	5
15	13	2100	2.75	6.5
17	14	2100	2.75	6.5
5	15	200	0.5	8
7	16	200	5	8
20	17	2100	2.75	6.5
8	18	4000	5	8
6	19	4000	0.5	8
4	20	4000	5	5

[1] There was an error during the execution of this run..

Starting GMMA having particle size of 51.6 nm (r. by dls) and OAg length of 30564 Da (by HPLC-SEC).

Response **% GMMA oxidation (HPAEC-PAD)**

Transform: **Square Root** **Constant:** **0.058**

These rows were ignored for this analysis.

7

ANOVA for Response Surface 2FI model

Analysis of variance table [Partial sum of squares - Type III]

**Source**	**Sum of Squares**	**df**	**Mean Square**	**F Value**	**p-value Prob > F**	
Model	47.598	6	7.933	210.558	1.9118E-11	*significant*
A-[GMMA]	8.914	1	8.914	236.594	2.915E-09	
B-[NaIO4]	31.388	1	31.388	833.093	1.8605E-12	
C-pH	2.791	1	2.791	74.071	1.7659E-06	
AB	0.536	1	0.536	14.224	0.00266495	
AC	2.640	1	2.640	70.073	2.3569E-06	
BC	1.330	1	1.330	35.297	6.8064E-05	
Residual	0.452	12	0.038			
Lack of Fit	0.220	7	0.031	0.677	0.692	*not significant*
Pure Error	0.232	5	0.046			
Cor Total	48.050	18				

Std. Dev.	0.194	R-Squared	0.991
Mean	2.965	Adj R-Squared	0.986
C.V. %	6.546	Pred R-Squared	0.963
PRESS	1.764	Adeq Precision	63.692
-2 Log Likelihood	-17.107	BIC	3.504
		AICc	7.075

Final Equation in Terms of Actual Factors:

Sqrt(% GMMA oxidation (HPAEC-PAD) + 0.06) =

4.487094	
-0.001669	* [GMMA]
1.699771	* [NaIO4]
-0.443261	* pH
-0.000061	* [GMMA] * [NaIO4]
0.000202	* [GMMA] * pH
-0.120804	* [NaIO4] * pH

(a)

**Response**	**OAg size (MP dRI)**

These rows were ignored for this analysis.
7

ANOVA for Response Surface Reduced 2FI model
Analysis of variance table [Partial sum of squares - Type III]

**Source**	**Sum of Squares**	**df**	**Mean Square**	**F Value**	**p-value Prob > F**	
Model	8.41E+08	4	2.10E+08	74.540	< 0.0001	*significant*
A-[GMMA]	8.29E+07	1	8.29E+07	29.370	< 0.0001	
B-[NaIO4]	7.16E+08	1	7.16E+08	253.860	< 0.0001	
C-pH	2.66E+07	1	2.66E+07	9.430	0.008	
AB	1.55E+07	1	1.55E+07	5.500	0.034	
Residual	3.95E+07	14	2.82E+06			
Lack of Fit	3.35E+07	9	3.72E+06	3.110	0.112	*not significant*
Pure Error	5.99E+06	5	1.20E+06			
Cor Total	8.81E+08	18				

Std. Dev.	1679.610	R-Squared	0.955
Mean	20888.950	Adj R-Squared	0.942
C.V. %	8.040	Pred R-Squared	0.898
PRESS	9.01E+07	Adeq Precision	30.493
-2 Log Likelihood	330.320	BIC	345.040
		AICc	344.930

Final Equation in Terms of Actual Factors:

OAg size (MP dRI) =
22521.574070
0.705910 * [GMMA]
-4445.173680 * [NaIO4]
1087.400000 * pH
0.325700 * [GMMA] * [NaIO4]

(b)

Figure S1. Identification of optimal conditions for GMMA oxidation: statistical analysis of the models for % GMMA oxidation (a) and OAg size (b)

Table S2. DoE approach applied to GMMA activation with BS3: summary of conditions tested.

Block	Run	Factor 1	Factor 2	Factor 3
		pH	[GMMA]	BS3 concentration (eq BS3 to NH_2 on GMMA)
			mg/mL	mg/mL (eq)
Day 1	1	6	0.2	1.2 (5)
Day 1	2	9	0.2	3.6 (15)
Day 1	3	7.5	2.1	25.2 (10)
Day 1	4	9	4	72.1 (15)
Day 1	5	9	0.2	1.2 (5)
Day 1	6	6	4	72.1 (15)
Day 1	7	6	4	24 (5)
Day 1	8	7.5	2.1	25.2 (10)
Day 1	9	7.5	2.1	25.2 (10)
Day 1	10	9	4	24 (5)
Day 1	11	6	0.2	3.6 (15)
Day 1	12	7.5	2.1	25.2 (10)
Day 2	13	7.5	2.1	12.6 (5)
Day 2	14	7.5	4	48.1 (10)
Day 2	15	6	2.1	25.2 (10)
Day 2	16	7.5	2.1	25.2 (10)
Day 2	17	7.5	2.1	37.8 (15)
Day 2	18	7.5	0.2	2.4 (10)
Day 2	19	9	2.1	25.2 (10)
Day 2	20	7.5	2.1	25.2 (10)

Response **% NH2 activation (TNBS)**

ANOVA for Response Surface Reduced Cubic model

Analysis of variance table [Partial sum of squares - Type III]

**Source**	**Sum of Squares**	**df**	**Mean Square**	**F Value**	**p-value Prob > F**	
Block	128.1687	1	128.1687011			
Model	1735.712	10	173.5711605	53.46789	3.15E-06	*significant*
A-pH	308.1359	1	308.1359333	94.92002	1.03E-05	
B-[GMMA]	738.1734	1	738.1733798	227.3913	3.7E-07	
C-eq BS3 to NH2 on GMMA	241.241	1	241.2409902	74.3133	2.54E-05	
AB	41.05079	1	41.05079148	12.64553	0.007443	
AC	0.074411	1	0.074411136	0.022922	0.883408	
BC	35.64661	1	35.6466111	10.98079	0.010639	
A^2	73.11089	1	73.11088939	22.52151	0.001453	
B^2	25.36879	1	25.36879225	7.814753	0.02336	
C^2	15.60412	1	15.60412436	4.806787	0.059694	
ABC	38.12519	1	38.12518624	11.74431	0.008995	
Residual	25.97015	8	3.246269198			
Lack of Fit	15.38771	4	3.846926685	1.454078	0.362811	*not significant*
Pure Error	10.58245	4	2.645611711			
Cor Total	1889.85	19				

Std. Dev.	1.801741	R-Squared	0.985258
Mean	51.63048	Adj R-Squared	0.966831
C.V. %	3.489684	Pred R-Squared	0.70962
PRESS	511.5576	Adeq Precision	30.43426
-2 Log Likelihood	61.98185	BIC	97.93064
		AICc	130.5533

Final Equation in Terms of Actual Factors:

% NH2 activation =	
-141.803	
40.1702	* pH
11.40516	* [GMMA]
4.953846	* eq BS3 to NH2 on GMMA
-0.73713	* pH * [GMMA]
-0.33457	* pH * eq BS3 to NH2 on GMMA
-0.92677	* [GMMA] * eq BS3 to NH2 on GMMA
-2.31953	* pH^2
-0.8516	* [GMMA]^2
-0.09644	* eq BS3 to NH2 on GMMA^2
0.153196	* pH * [GMMA] * eq BS3 to NH2 on GMMA

(a)

Response **% active ester groups (HPLC-SEC)**

These rows were ignored for this analysis.
10

ANOVA for Response Surface Reduced Quadratic model
Analysis of variance table [Partial sum of squares - Type III]

**Source**	**Sum of Squares**	**df**	**Mean Square**	**F Value**	**p-value Prob > F**	
Block	328.6142	1	328.6141536			
Model	673.5975	5	134.7195007	9.402123	0.000778	*significant*
A-pH	12.15521	1	12.15521342	0.848317	0.375175	
B-[GMMA]	225.14	1	225.1400244	15.7126	0.00188	
C-eq BS3 to NH2 on GMMA	244.837	1	244.8370265	17.08727	0.001386	
A^2	126.9122	1	126.9122129	8.85725	0.011569	
C^2	165.231	1	165.2309933	11.53153	0.005311	
Residual	171.9435	12	14.32862531			
Lack of Fit	129.8367	8	16.22958835	1.541755	0.356404	*not significant*
Pure Error	42.1068	4	10.52669924			
Cor Total	1174.155	18				

Std. Dev.	3.785317	R-Squared	0.796647
Mean	19.33909	Adj R-Squared	0.711916
C.V. %	19.5734	Pred R-Squared	0.45243
PRESS	462.9929	Adeq Precision	11.9817
-2 Log Likelihood	95.77148	BIC	116.3825
		AICc	119.9533

Final Equation in Terms of Actual Factors:

gruppi reattivi / GMMA	
143.4041	
-44.1813	* pH
2.703501	* [GMMA]
7.011795	* eq BS3 to NH2 on GMMA
2.892371	* pH^2
-0.29702	* eq BS3 to NH2 on GMMA^2

(b)

Figure S2. Identification of optimal conditions for GMMA derivatization with BS3: statistical analysis of the models for % NH_2 activation (a) and % active ester groups introduced (b). Row 10 was ignored in (b) as the sample was erroneously poured out before analysis.

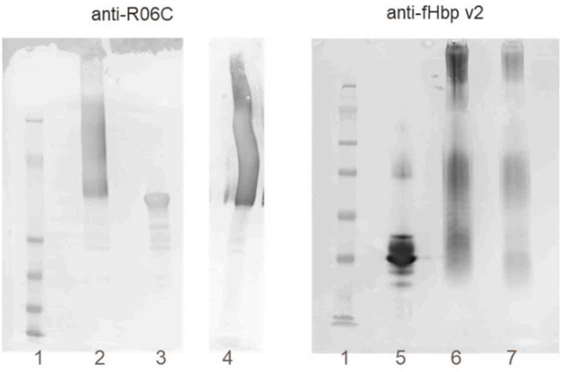

Figure S3. Characterization by WB analysis of GMMA-R06C and GMMA-fHbp v2 conjugates produced by reductive amination and BS3 chemistries respectively in comparison to corresponding unconjugated proteins. Ten μg of conjugates and 2 μg of protein were loaded per well. Lane 1: marker, lane 2: STm GMMAox-R06C recycled, lane 3: R06C, lane 4: STm GMMAox-R06C, lane 5: fHbp v2, lane 6: STm GMMA-BS3-fHbp v2, lane 7: STm GMMA-BS3-fHbp v2 recycled.

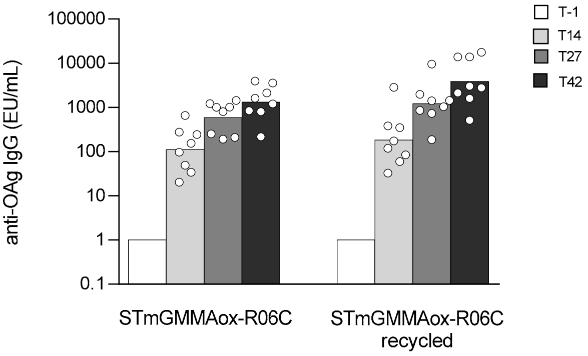

Figure S4. Immunogenicity of R06C-GMMA conjugates (reductive amination chemistry) in mice. CD1 mice were immunized subcutaneously at day 0 and 28 with 36 μg and 56 μg total protein dose (corresponding to 4 μg R06C/dose) for the R06C non-recycled and recycled conjugates respectively. All constructs were formulated with 0.7 mg/mL Alhydrogel. Sera were analyzed at days -1, 14, 27 and 42 by ELISA using as coating antigen STm OAg. Summary graphs of anti-antigen specific IgG geometric mean units (bars) and individual antibody levels (dots) are reported.

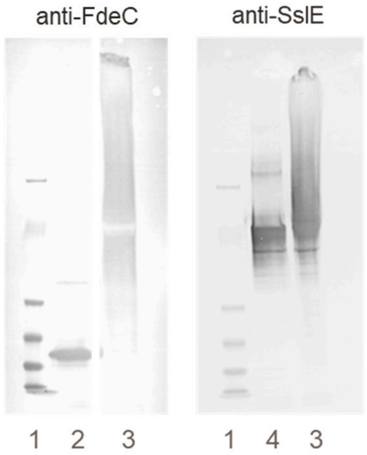

(a)

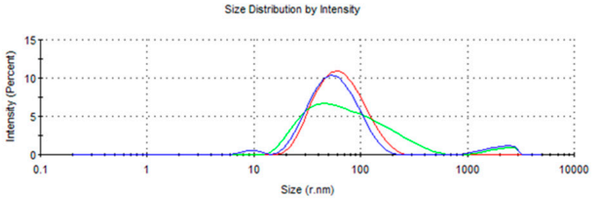

(b)

Figure S5. Bivalent conjugate characterization by WB and dls analyses. (a) WB analysis of the conjugate in comparison to corresponding unconjugated proteins. Ten μg of conjugate and 2 μg of proteins were loaded per well. Lane 1: marker, lane 2: FdeC, lane 3: orthogonal bivalent conjugate, lane 4: SslE. (b) dls analysis of *S. sonnei* GMMA (red line, Z average r of 56.3 nm, PdI of 0.197), *S. sonnei* GMMA-BS3 (green line, Z average r of 58.1 nm, PdI of 0.409) and bivalent conjugate (blue line, Z average r of 51.4 nm, PdI of 0.316), confirming no crosslinking after conjugation.

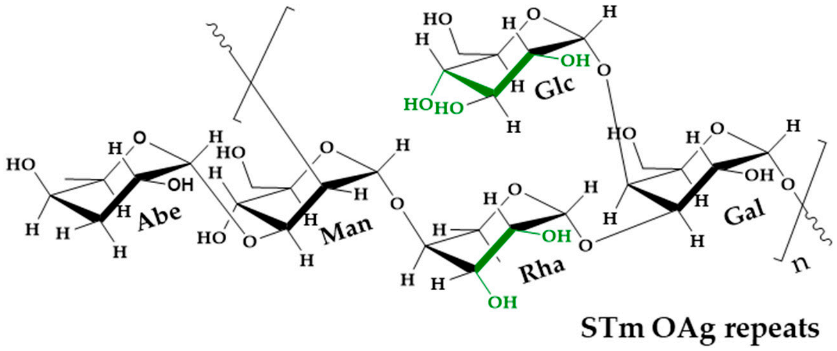

Figure S6. Vicinal diols, in STm GMMA OAg, which are susceptible to oxidation with $NaIO_4$: present at the Rha and Glc residues, highlighted in green.
